# 靶向CD6的新型CAR-T细胞治疗急性T淋巴细胞白血病：一种通过基因编辑消除自相残杀的安全高效策略

**DOI:** 10.3760/cma.j.cn121090-20260130-00068

**Published:** 2026-05

**Authors:** 杨韬 上官, 乐灵 谢, 文兵 刘, 闰夏 顾, 颖茜 徐, 敏 王, 建祥 王

**Affiliations:** 1 中国医学科学院血液病医院（中国医学科学院血液学研究所），血液与健康全国重点实验室，国家血液系统疾病临床医学研究中心，细胞生态海河实验室，天津 300020 State Key Laboratory of Experimental Hematology, National Clinical Research Center for Blood Diseases, Haihe Laboratory of Cell Ecosystem, Institute of Hematology & Blood Diseases Hospital, Chinese Academy of Medical Sciences & Peking Union Medical College, Tianjin 300020, China; 2 天津医学健康研究院，天津 301600 Tianjin Institutes of Health Science, Tianjin 301600, China

**Keywords:** 白血病，T淋巴细胞，急性, 嵌合抗原受体T细胞, CD6, 基因编辑, Leukemia, T lymphoblastic, acute, Chimeric antigen receptor T-cell, CD6, Gene editing

## Abstract

**目的:**

探索克服急性T淋巴细胞白血病（T-ALL）嵌合抗原受体T细胞（CAR-T细胞）疗法中自相残杀与非肿瘤靶向毒性双重障碍的新策略，通过鉴定新靶点及其相应基因编辑方案，开发一种安全、高效的抗T-ALL CAR-T产品。

**方法:**

①利用公共数据库单细胞转录组测序（scRNA-seq）数据分析T-ALL患者及健康供者骨髓细胞，评估CD6与CD7的表达谱差异。②为探究CD6在CAR-T细胞中的内源性作用，首先采用CRISPR/Cas9 RNP系统在CD19 CAR-T模型中，评估CD6敲除对CAR-T细胞表型和活化状态的影响；随后，构建CD6敲除的靶向CD6 CAR-T细胞（6KO-6CAR）并评估其功能。

**结果:**

scRNA-seq数据显示，CD6在T-ALL中广泛表达，且相较于在部分正常造血干/祖细胞及髓系细胞中均表达的传统靶点CD7，CD6的表达谱更为局限，展现出更优的安全性特征。CD19 CAR-T模型中的研究表明，CD6敲除能使CAR-T细胞维持更优的功能状态：其基线活化水平（CD25表达）降低（*P*<0.05），同时在抗原刺激下能产生更高比例的TNF-α^+^IFN-γ^+^细胞（*P*<0.05）。进一步构建的6KO-6CAR细胞在体外对多种CD6^+^ T-ALL细胞系（MOLT-4、CCRF-CEM、Jurkat）表现出较强的特异性激活（CD107a表达显著上调，均*P*<0.001）及杀伤作用（均*P*<0.05）。

**结论:**

CD6是一个具有高覆盖性和良好安全性的T-ALL治疗新靶点，同时敲除内源性CD6后还能从整体上优化CAR-T细胞的内在功能状态。基于CRISPR/Cas9技术构建6KO-6CAR的策略，能在解决CAR-T细胞自相残杀的同时增强其抗肿瘤功能，这为T-ALL提供了一种兼具安全性与应用前景的新型免疫治疗方案。

## Abstract (en)

**Objective:**

To explore a novel strategy that addresses the dual challenges of fratricide and on-target off-tumor toxicity in current chimeric antigen receptor T-cell（CAR-T）therapy for T-cell acute lymphoblastic leukemia（T-ALL）and to develop a safe and efficacious anti-T-ALL CAR-T product by identifying a new target and compatible gene-editing approach.

**Methods:**

Public single-cell RNA sequencing（scRNA-seq）datasets were utilized to analyze bone marrow cells extracted from patients with T-ALL and healthy donors, evaluating the differential expression profiles of CD6 and CD7. In investigating the endogenous role of CD6 in CAR-T cells, the CRISPR/Cas9 RNP system was first employed in a CD19 CAR-T model to evaluate the impact of CD6 knockout on the phenotype and activation status of CAR-T cells. Subsequently, CD6-knockout, CD6-targeted CAR-T cells（6KO-6CAR）were constructed, and their functional activities were evaluated.

**Results:**

scRNA-seq analysis revealed that CD6 is broadly expressed in T-ALL. Compared with the traditional target CD7, which is also expressed in a subset of normal hematopoietic stem/progenitor cells and myeloid cells, CD6 exhibits a more restricted expression profile, exhibiting superior safety characteristics. Studies on the CD19 CAR-T model indicated that CD6 knockout enables CAR-T cells to maintain a superior functional state: their baseline activation level（CD25 expression）was reduced（*P*<0.05）while generating a higher proportion of TNF-α^+^IFN-γ^+^ cells（*P*<0.05）upon antigen stimulation. The further constructed 6KO-6CAR cells exhibited potent specific activation（significantly upregulated CD107a expression level, all *P*<0.001）and cytotoxicity（all *P*<0.05）against multiple CD6^+^ T-ALL cell lines（MOLT-4, CCRF-CEM, and Jurkat）in vitro.

**Conclusion:**

CD6 is a novel therapeutic target for T-ALL with high coverage and a favorable safety profile, and knocking out endogenous CD6 globally optimizes the intrinsic functional state of CAR-T cells. Constructing 6KO-6CAR based on the CRISPR/Cas9 technology addresses fratricide in CAR-T cells while enhancing their antitumor functionality, thereby providing a novel immunotherapy regimen with safety and clinical translational potential for relapsed/refractory T-ALL.

急性T淋巴细胞白血病（T-ALL）是一种侵袭性强、进展迅速的血液系统恶性肿瘤。尽管联合化疗方案的应用提高了初始缓解率，但复发/难治性（r/r）T-ALL患者的预后依然极差，临床亟需更有效的治疗手段[1]。近年来，以靶向CD19为代表的嵌合抗原受体T细胞（CAR-T细胞）疗法在B细胞恶性肿瘤中取得了巨大成功，但其在T-ALL中的应用仍然面临严峻挑战[2]–[3]。核心瓶颈在于，大多数T细胞相关抗原（如CD7、CD5等）在肿瘤细胞与正常T细胞上共表达，导致CAR-T细胞在制备过程中发生自相残杀，难以扩增[4]–[9]；同时，这些靶点在正常造血干/祖细胞（HSPC）等关键群体上的表达还会引发严重的非肿瘤靶向毒性，造成长期骨髓抑制与免疫缺陷[10]。因此，寻找一个兼顾广谱肿瘤覆盖与安全性的新靶点，并开发相应的策略以克服自相残杀效应和优化CAR-T细胞功能，是T-ALL免疫治疗领域的关键科学问题。

随着单细胞转录组学技术的发展，全面解析骨髓微环境中免疫细胞的抗原表达并精准筛选肿瘤特异性靶点成为了可能。本研究发现CD6可以作为T-ALL的新型候选靶点。与现有疗法相比，靶向CD6有望规避CD7 CAR-T引发的骨髓抑制风险，提升细胞免疫疗法的安全性。为克服潜在的制备障碍，本研究采取了在制备CD6 CAR-T的同时，同步敲除其内源性CD6基因的策略。更为关键的是，本研究不仅将CD6敲除作为制备过程，还进一步揭示了该基因编辑对CAR-T细胞功能表型与效应潜力的影响，为开发安全、高效的T-ALL免疫治疗产品提供了新思路。

## 材料与方法

一、材料与试剂

1. 细胞株与临床样本：Jurkat、MOLT-4、CCRF-CEM、Nalm-6及HEK-293T细胞系均由本实验室保存。本研究已获中国医学科学院血液病医院（中国医学科学院血液学研究所）伦理审查委员会的批准（批件号：NSFC2023115-EC-2）。健康供者外周血源自天津市中心血站；T-ALL患者外周血单个核细胞（PBMC）和骨髓单个核细胞来源于本院住院患者，所有样本提供者均已签署知情同意书。

2. 质粒与试剂：慢病毒包装质粒（pMD2.G、pRSV-Rev、pMDLg/pRRE）及CD19-CAR、CD6-CAR目的质粒（两种目的质粒均包含GFP序列）由本实验室构建并保存。CD6靶向和非靶向（NT）gRNA序列由苏州金唯智生物科技有限公司合成；Cas9重组蛋白购自美国IDT公司；RosetteSep人T细胞富集抗体购自加拿大STEMCELL公司；人原代T细胞活化磁珠Dynabeads™ CD3/CD28购自美国Thermo Fisher公司；KBM581淋巴细胞培养基购自美国Corning公司；DMEM和RPMI1640培养基均购自于美国Gibco公司；重组人IL-2购自美国PeproTech公司；DiD细胞膜染料购自上海爱必信公司；Ficoll密度梯度分离液购自天津灏洋生物制品科技有限公司；4D-Nucleofector电转仪及P3 Primary Cell电转试剂盒购自瑞士Lonza公司；APC-兔抗鼠IgG F（ab'）_2_抗体购自美国Jackson Immuno Research公司；抗人P53抗体购自美国Santa Cruz公司；抗人GAPDH抗体购自武汉赛维尔生物科技有限公司；辣根过氧化物酶标记（HRP）的山羊抗小鼠IgG和山羊抗兔IgG均购自北京中杉金桥生物技术有限公司；RIPA蛋白裂解液、QuickBlock™ Western一抗稀释液、脱脂奶粉均购自上海碧云天生物科技有限公司；苯甲基磺酰氟（PMSF）购自美国Roche公司；ECL化学发光液购自南京诺唯赞生物科技股份有限公司；FastPAGE蛋白预制胶购自北京擎科生物科技股份有限公司；PE-抗人CD6抗体、APC-抗人CD3抗体、PE-抗人CD25抗体、PE/Cy7-抗人CD107a抗体、APC/Cy7-抗人IFN-γ抗体、PE-抗人TNF-α抗体、同型对照抗体、细胞激活试剂（Cell activation cocktail with Brefeldin A）、莫能霉素及细胞死活染色试剂（LIVE/DEAD™ Fixable Violet）均购自美国BioLegend公司；固定/破膜试剂盒（Cytofix/Cytoperm™ Fixation and Permeabilization Solution）购自美国BD公司。

3. 细胞培养：所有细胞均置于37 °C、5％CO_2_、饱和湿度的细胞培养箱中常规传代培养，取对数生长期细胞用于后续实验。Jurkat、MOLT-4、CCRF-CEM、Nalm-6细胞培养于含10％胎牛血清的RPMI 1640培养基中。HEK-293T细胞培养于含10％胎牛血清的DMEM培养基中。人原代T细胞培养于含5％胎牛血清和50 U/ml IL-2的KBM581培养基中。

二、实验方法

1. 数据分析：从单细胞公共数据库下载数据集SCPCP000003（https://scpca.alexslemonade.org/projects/SCPCP000003）、GSE120221和HRA014168，以获得基因-细胞表达矩阵。所有单细胞转录组数据在R中采用Seurat 4.4.0包进行分析。质控过滤标准为：每个细胞检测到的基因数量介于300～7 000，且线粒体基因比例低于15％。过滤后，采用LogNormalize方法对表达矩阵进行标准化，并通过FindVariableFeatures函数筛选高变基因。基于高变基因进行主成分分析降维，并基于显著主成分进行细胞聚类，使用UMAP（Uniform Manifold Approximation and Projection）方法进行可视化展示及细胞亚群注释。利用FeaturePlot和DotPlot分别展示SCPCP000003和GSE120221数据集中CD6、CD7在不同细胞亚群中的表达分布及表达水平。对HRA014168中的靶向CD19 CAR-T单细胞转录组数据使用scTenifoldKnk（https://github.com/cailab-tamu/scTenifoldKnk）进行虚拟基因敲除分析，以预测目标基因缺失后可能引起的下游转录调控变化。对筛选得到的潜在调控基因集合（筛选阈值设定为*P*<0.05）进一步进行Gene Ontology（GO）和Kyoto Encyclopedia of Genes and Genomes（KEGG）通路富集分析，以探讨相关基因的生物学功能及潜在作用通路。

2. 流式细胞检测患者骨髓单个核细胞中CD6表达：收集T-ALL患者的EDTA抗凝处理的穿刺骨髓液或外周血3 ml，经Ficoll密度梯度分离，加入PE标记的抗人CD6抗体，室温避光孵育20 min，经PBS洗涤重悬后使用安捷伦NovoCyte 3000流式细胞分析仪进行检测，使用NovoExpress软件进行分析。

3. 慢病毒包装与浓缩：取对数生长期HEK-293T细胞接种于10 cm培养皿，待汇合度达90％时，将包装质粒体系（3.5 µg pMD2.G、2.56 µg pRSV-Rev、5.33 µg pMDLg/pRRE及16 µg目的质粒）与1 µg/µl PEI溶液混合，室温静置12 min后均匀滴加至细胞培养液中。转染12～16 h后更换为含20％胎牛血清的DMEM培养基，48 h后收集病毒上清。经0.45 µm滤膜过滤后，超速离心（50 000×*g*，2.5 h，4 °C）浓缩病毒液，分装保存于−80 °C备用。

4. CAR-T细胞的制备及基因编辑：采用RosetteSep抗体复合物联合Ficoll密度梯度离心法分离人外周血T细胞。细胞重悬于T细胞完全培养基中，按磁珠与细胞1∶1的比例加入Dynabeads™ CD3/CD28磁珠激活（记为第0天）。于第2天去除磁珠，收集T细胞。将gRNA与Cas9蛋白按3∶1摩尔比混合，37 °C孵育5 min以组装核糖核蛋白复合物（RNP）。利用P3试剂盒和Lonza 4D电转系统（程序EO-115）进行电转。静置恢复4 h后加入浓缩的CD6-CAR或CD19-CAR慢病毒液进行转导，常规培养扩增至第8～9天用于后续功能实验。

5. CAR-T细胞扩增倍数检测：将磁珠激活（第0天）时记为初始倍数，然后在各时间点检测T细胞的数量，计算扩增倍数。相对扩增提高率（％）通过以下公式计算：［（实验组细胞扩增倍数−对照组细胞扩增倍数）/对照组细胞扩增倍数］×100％。

6. 流式细胞术检测CAR修饰T细胞的阳性率：T细胞转导后96 h，用APC-兔抗鼠IgG F（ab'）_2_抗体标记CAR-T细胞，通过流式细胞术同时检测GFP和Fab的表达，分析转导效率。

7. CD19 CAR-T体外活化评估：使用抗CD25单克隆抗体进行标记，使用流式细胞术检测T细胞中CD25表达的比例。

8. CD19 CAR-T体外杀伤功能评估：选取CD19^+^ B-ALL细胞系Nalm-6作为靶细胞，将效应T细胞与靶细胞按效靶比1∶4混合，置于无IL-2培养基中共培养。24 h后收集细胞，使用CD3抗体标记，随后流式细胞术检测残余活靶细胞（CD3⁻）的比例和绝对数。

9. CD19 CAR-T胞内细胞因子检测：取各组5×10^5^效应CD19 CAR-T细胞接种于24孔板，加入1×细胞激活试剂，于37 °C、5％ CO_2_培养箱中孵育4 h。收集细胞，经死活细胞染色、固定破膜后，使用抗人IFN-γ及TNF-α流式抗体于4 °C避光染色30 min。使用流式细胞术检测活细胞中IFN-γ及TNF-α的表达水平。

10. CD6 CAR-T体外杀伤功能：选取CD6^+^ T-ALL细胞系（Jurkat、MOLT-4、CCRF-CEM等）作为靶细胞，使用5 mmol/L DiD染料避光染色20 min。将效应T细胞与靶细胞按效靶比1∶1、5∶1、10∶1混合，置于无IL-2培养基中共培养。24 h后收集细胞，使用流式细胞术检测残余活靶细胞（DiD^+^）的比例和绝对数。

11. CD6 CAR-T脱颗粒功能检测：按效靶比1∶1混合效应T细胞与DiD标记的靶细胞，加入PE/Cy7标记的抗人CD107a抗体及50 U/ml IL-2，37 °C共孵育1 h后加入莫能霉素，继续培养4 h。流式细胞术检测DiD⁻效应细胞群中CD107a^+^细胞比例。

12. Western blot检测P53蛋白表达：实验分为CD6未敲除组（Non-Targeting KO，NT）和CD6敲除组（CD6-KO gCD6）组。T细胞经抗CD3/CD28磁珠刺激活化24 h后，通过BD FACSAria Ⅲ流式分选仪分选获得活细胞。收集的细胞沉淀中加入含有蛋白酶抑制剂（PMSF）的RIPA裂解液，冰上裂解30 min。裂解液经离心（4 °C，12 000×*g*，5 min）取上清，加入5× SDS-PAGE上样缓冲液并于100 °C煮沸5 min使蛋白变性。取等量蛋白样品经SDS-PAGE预制胶电泳分离，并转移至PVDF膜。膜经5％脱脂牛奶室温封闭1 h，加入一抗于4 °C孵育过夜。洗涤后，加入相应HRP标记的二抗室温孵育1 h，使用ECL发光显影。利用ImageJ软件进行蛋白定量分析，以GAPDH为内参进行归一化处理。

三、统计学处理

使用GraphPad Prism 10.1.2软件进行统计学分析，所有实验均至少重复3次，数据以均数±标准差表示。对于完全随机设计的两组间比较，采用独立样本*t*检验；对于双因素设计的多组间比较，采用双因素方差分析（Two-way ANOVA）。双侧*P*<0.05为差异有统计学意义。

## 结果

一、单细胞转录组测序与流式细胞术评估CD6作为T-ALL治疗靶点的可行性

为筛选潜在的T-ALL免疫治疗靶点，我们利用SCPCP000003数据集中的T-ALL患者单细胞转录组测序（scRNA-seq）数据，系统分析了其骨髓免疫细胞的抗原表达谱。UMAP聚类结果显示肿瘤细胞具有高度的克隆异质性（C1～C8亚群），同时CD6抗原在这些不同的白血病亚克隆及正常T细胞中均呈广泛性高表达（图1A、B）。流式细胞术检测进一步证实6例T-ALL患者的原代白血病细胞均一致性地高表达CD6（图1C）。

**图1:**
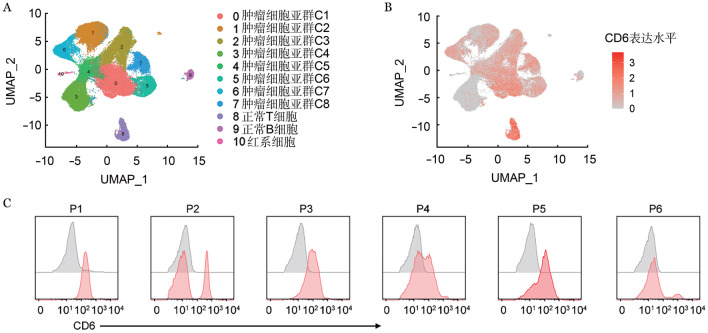
基于单细胞转录组测序与流式细胞术急性T淋巴细胞白血病（T-ALL）细胞的CD6表达特征评价 **A** UMAP聚类展示T-ALL细胞高度的克隆异质性（C1～C8亚群）；**B** UMAP图展示CD6抗原在各白血病亚克隆及正常T细胞中的表达水平；**C** 流式细胞术检测6例T-ALL患者（P1～P6）原代白血病细胞表面CD6的表达情况

靶向非肿瘤毒性的评估是新型CAR-T疗法的重要环节。为此，我们进一步分析了GSE120221数据集中健康供者骨髓的scRNA-seq数据（图2A），发现常用的恶性T细胞血液肿瘤靶点CD7在T细胞、NK细胞及髓系祖细胞中均有广泛表达（图2B）。相比之下，CD6的表达谱更为局限，主要集中于T细胞及部分NK细胞亚群，而在红系细胞、B细胞、单核细胞及HSPC中几乎不能检测到CD6表达（图2C）。上述结果表明，选择CD6作为靶点不仅能通过CAR-T细胞实现对T-ALL的广谱覆盖，还能最大程度避免对正常造血系统（如HSPC）的影响。

**图2:**
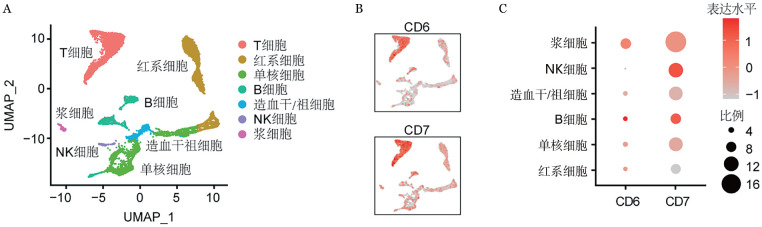
CD6与CD7在正常造血系统中的表达谱对比分析 **A** 健康供者骨髓样本的单细胞转录组测序UMAP聚类图；**B** UMAP图展示CD6、CD7基因在正常造血系统中的表达分布；**C** 气泡图展示CD6与CD7在主要非T系细胞及造血干/祖细胞中的表达

二、CD6基因敲除体系的建立及其对CAR-T细胞功能的影响

与CD7相似，正常T细胞也表达较高水平的CD6（图1B和图2B）。为了避免靶向CD6 CAR-T的自相残杀效应，我们首先建立并验证了CD6基因编辑体系的有效性。流式细胞术结果证实，CRISPR/Cas9 RNP系统能够高效消除T细胞表面的CD6表达（图3A）。在此基础上，我们发现，与NT组相比，gCD6组CAR的表达水平显著增加（图3B）。同时细胞体外扩增计数表明，在培养至第6天时，gCD6组CD6 CAR-T细胞的相对扩增倍数显著高于NT组，平均提高约76％（图3C）。

**图3:**
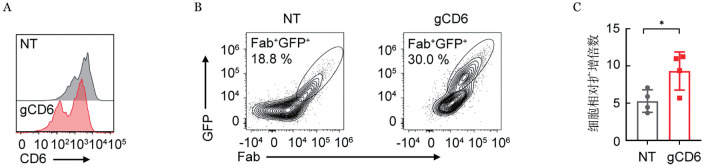
CD6基因敲除及抗CD6嵌合抗原受体T细胞（CAR-T细胞）的构建与验证（*P<0.05） **A** 流式细胞术检测CRISPR/Cas9 RNP系统编辑后的T细胞表面CD6表达情况；**B** 流式细胞术检测CD6未敲除组（NT）与CD6敲除组（gCD6）CD6 CAR-T中的CAR阳性T细胞比例；**C** 培养第6天，NT与gCD6组CD6 CAR-T细胞的相对扩增倍数（*n*＝4）

为避免CD6 CAR-T中自相残杀效应对功能验证的干扰，从而无法直接解析CD6的作用，我们采用靶向CD19的CAR-T模型，系统研究CD6敲除对CAR-T细胞生物学功能的影响。首先利用scTenifoldKnk算法对来源于HRA014168公共数据集的靶向CD19 CAR-T单细胞数据进行了CD6基因的模拟敲除分析。GO和KEGG富集分析结果显示，CD6的缺失显著影响了多条与T细胞功能密切相关的生物学过程和信号通路，其中包括“T细胞激活调控”和“细胞杀伤”等关键功能模块，NF-κB和P53等经典信号通路也在差异基因中显著富集（图4A）。为了进一步验证生信预测的可靠性，我们探究了CD6对T细胞内关键信号分子的影响。实验结果显示，在TCR信号刺激下，CD6敲除显著下调了T细胞中P53的表达水平，平均降低了26％（图4B）。这一分子层面的证据提示CD6可能参与调控T细胞的活化及功能信号网络调控。体外功能实验显示，尽管在敲除CD6之后，CD19 CAR-T的体外扩增能力未受明显影响（图4C），在无抗原刺激的培养条件下，未敲除CD6对照组的CAR-T细胞表现出较高水平的CD25表达，可能存在基线活化信号导致的早期耗竭（图4D），提示CD6缺失可能重塑了T细胞的活化阈值，使其维持在更具潜能的静息状态。功能学实验进一步证实，在受到靶抗原刺激时，gCD6 CAR-T可以产生更高比例的TNF-α^+^IFN-γ^+^多能性T细胞（图4E）。特别是在模拟体内高肿瘤负荷的体外“压力测试”（效靶比1∶4）中，gCD6 CAR-T能维持较高的细胞毒活性（图4F）。上述结果证明，CD6基因敲除不仅在制备阶段能提升CAR表达水平，并且能通过优化转录调控网络，显著增强CAR-T细胞的抗肿瘤潜能。

**图4:**
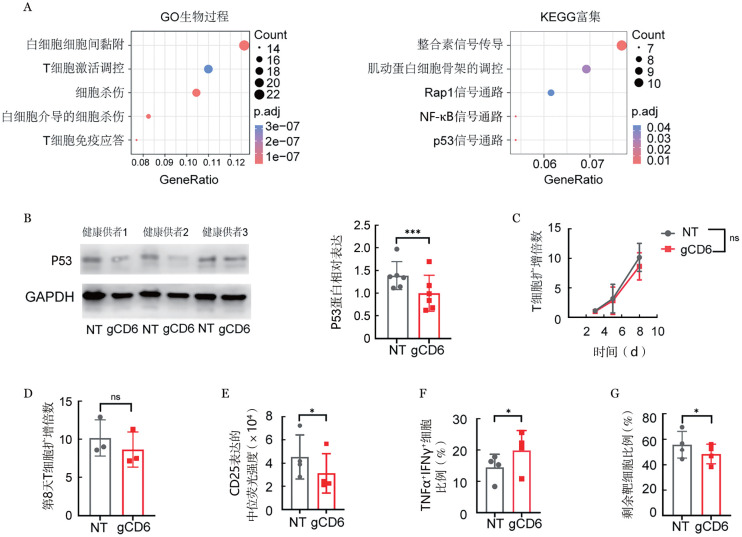
CD6基因敲除对p53信号通路及CD19嵌合抗原受体T细胞（CAR-T细胞）功能的影响（*P<0.05、**P<0.01、***P<0.001，ns差异无统计学意义） **A** 利用scTenifoldKnk算法模拟CD19 CAR-T敲除CD6基因后的单细胞转录组变化，GO与KEGG富集分析CD6缺失主要影响的转录调控网络；**B** Western blot检测在TCR信号刺激后NT与gCD6组T细胞中P53表达水平（*n*＝6）；**C** NT组与gCD6组CD19 CAR-T细胞体外扩增曲线（*n*＝3）；**D** NT组与gCD6组CD19 CAR-T细胞第8天体外扩增倍数比较（*n*＝3）；**E** 无抗原刺激条件下，NT组与gCD6组CD19 CAR-T细胞表面CD25表达水平（*n*＝4）；**F** 靶抗原刺激后CAR-T细胞中TNFα^+^IFNγ^+^双阳性T细胞比例（*n*＝4）；**G** 效靶比1∶4下，CD19 CAR-T杀伤后的剩余靶细胞比例（*n*＝4） **注** NT：CD6未敲除组；gCD6：CD6敲除组

三、基于CD6敲除策略构建针对T-ALL的靶向CD6 CAR-T细胞

基于上述发现，我们将“CD6靶点的高特异性”与“CD6敲除的增效机制”相结合，构建了CD6基因敲除的靶向CD6 CAR-T细胞（6KO-6CAR），并验证其抗白血病细胞的能力。我们选取了3种CD6^+^ T-ALL细胞系（MOLT-4、CCRF-CEM、Jurkat）作为靶细胞（图5A）。CD107a脱颗粒实验显示，与同样经CD6敲除但转导空载体的对照T细胞（6KO-Vec）相比，6KO-6CAR细胞在识别肿瘤抗原后发生了较强的特异性激活，表现为CD107a阳性率显著上调（图5B）。杀伤实验显示，6KO-6CAR细胞对不同T-ALL细胞均表现出有效的杀伤作用（图5C、D）。这一系列结果显示，我们研发的6KO-6CAR策略成功克服了自相残杀障碍，并具备良好的抗T-ALL活性。

**图5:**
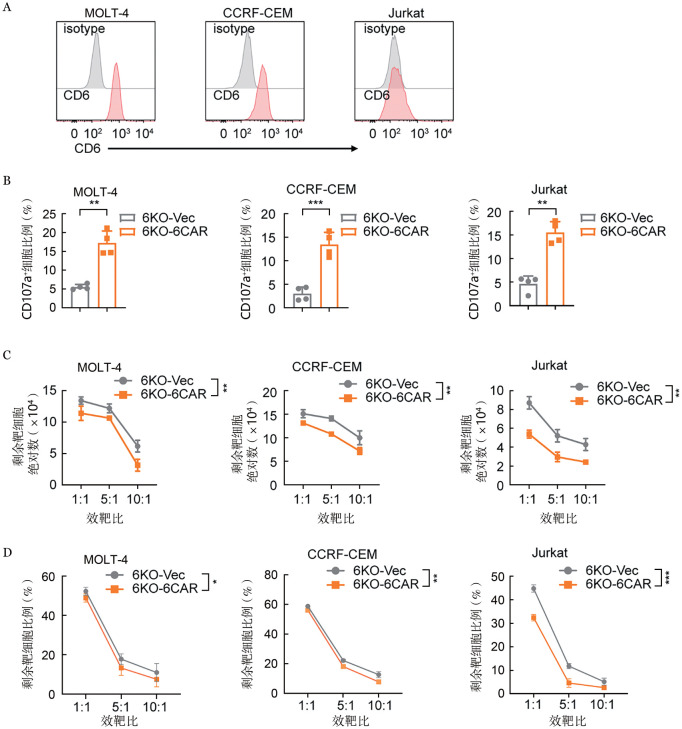
CD6 KO-6CAR对3种T-ALL细胞系（MOLT-4、CCRF-CEM、Jurkat）的杀伤能力评估（*P<0.05、**P<0.01、***P<0.001） **A** 流式细胞术检测T-ALL细胞表面CD6的表达情况；**B** 流式细胞术检测6KO-Vec和6KO-6CAR效应细胞中CD107a的表达水平（*n*＝4）；**C** 与6KO-6CAR细胞共培养后T-ALL细胞的剩余靶细胞数（*n*＝3）；**D** 与6KO-6CAR细胞共培养后T-ALL细胞的剩余靶细胞比例（*n*＝3） **注** 6KO-6CAR：CD6基因敲除的靶向CD6 CAR-T细胞；6KO-Vec：CD6敲除并转导空载体的对照T细胞

## 讨论

CAR-T疗法在r/r T-ALL的治疗中仍面临巨大挑战，核心障碍在于CAR-T细胞与肿瘤细胞共享表面抗原（如CD7、CD5等），导致严重的自相残杀效应，极大地限制了CAR-T细胞在体外的有效扩增与杀伤功能的维持[4]–[5]。本研究通过CRISPR/Cas9技术，构建了内源性CD6基因敲除的靶向CD6 CAR-T细胞（6KO-6CAR），不仅从技术上克服了自相残杀难题，更揭示了CD6作为治疗靶点在安全性与功能调节方面的独特优势。

T-ALL免疫治疗方案的核心重点是靶点的安全性评估。近年来在临床试验中受到广泛关注的CD7靶点虽疗效显著，但CD7分子广泛表达于正常T细胞、NK细胞及部分髓系前体细胞[11]。多项临床研究表明，CD7 CAR-T治疗常导致严重的靶向非肿瘤毒性，引发长期的T/NK细胞再生障碍及潜在的髓系抑制，导致患者往往面临极高的感染风险，且通常需要桥接异基因造血干细胞移植以重建免疫系统[12]–[13]。相比之下，本研究对单细胞转录组测序数据进行分析后发现，CD6主要表达于成熟T细胞及部分NK细胞亚群，而在HSPC、粒系及红系细胞中几乎不表达。这一特征表明靶向CD6的CAR-T疗法有望在发挥抗白血病效应的同时，显著降低对患者骨髓造血系统及部分天然免疫功能的损伤风险，在安全性方面展现出优于现有CD7靶点的临床转化潜力。

除了作为表面靶点，CD6分子对T细胞受体（TCR）信号的动态调控作用也是本研究关注的重点。CD6属于清道夫受体富含半胱氨酸（scavenger receptor cysteine-rich, SRCR）超家族成员，传统观点认为其通过结合ALCAM（CD166）提供共刺激信号。然而，有研究指出，CD6实际上是一个信号变阻器（Rheostat），其胞内长尾结构可招募多种参与TCR信号转导的接头蛋白（如GADS、SLP-76等），并通过调节信号复合体的组装对T细胞活化反应进行微调[14]。在本研究中，首先通过单细胞数据模拟敲除发现CD6会影响NF-κB和p53信号轴。既往研究表明，该信号轴在T细胞的激活、增殖及效应功能调控中发挥重要作用，因此这些通路的富集变化从机制层面支持了CD6对T细胞信号转导的调控作用[15]–[17]。随后的实验证明敲除CD6不仅防止了自相残杀，还显著降低了P53蛋白的表达和CAR-T细胞在无抗原刺激下的基线CD25表达水平（图4）。由于P53在介导细胞应激、凋亡及耗竭中发挥核心作用[18]–[19]，这提示在CAR-T的高密度培养体系中，CD6可能介导了T细胞的基线活化，导致T细胞过早耗竭。通过基因编辑敲除CD6，在阻断自相残杀的同时，解除了这种慢性刺激，使CAR-T细胞维持在更佳的静息记忆状态，提示其在面对高肿瘤负荷时，可能具备更持久的抗肿瘤潜力，但仍需进一步的实验验证。

尽管本研究在体外实验中显示了抗CD6 CAR-T疗法清除T-ALL的可行性，但其抗原识别结构域（scFv）的亲和力仍需进一步优化。我们目前使用的单链抗体可变区基因片段序列源自治疗自身免疫性疾病的专利（CN112638478A）。值得注意的是，该序列最初是为CAR-Treg细胞设计，旨在发挥免疫调节功能以缓解自身免疫反应，而非介导效应T细胞对肿瘤的直接杀伤。既往研究表明scFv的亲和力并非越高越好，而是需要经过亲和力优化和筛选，以在高效杀伤与避免耗竭之间取得最佳平衡[20]。鉴于该专利序列并未针对肿瘤清除的动力学特征进行过优化，其与效应T细胞杀伤模式的潜在不匹配可能是限制本研究中抗CD6 CAR-T细胞杀伤效力的主要原因之一。

综上所述，本研究表明了基于CRISPR/Cas9敲除CD6的策略能够成功构建高效、特异的抗CD6 CAR-T细胞。该策略不仅能有效克服T-ALL患者CAR-T制备中自相残杀的技术瓶颈，还借助了内源性敲除CD6对T细胞的功能增效，为r/r T-ALL患者提供了一种较CD7靶点更为安全的免疫治疗新方案。

## References

[1] Ribera JM, Morgades M, Genescà E, on behalf of the PETHEMA Group (2021). Outcomes and prognostic factors of adults with refractory or relapsed T-cell acute lymphoblastic leukemia included in measurable residual disease-oriented trials[J]. Hematol Oncol.

[2] Vose J, Armitage J, Weisenburger D (2008). International peripheral T-cell and natural killer/T-cell lymphoma study: pathology findings and clinical outcomes[J]. J Clin Oncol.

[3] Maciocia PM, Wawrzyniecka PA, Philip B (2017). Targeting the T cell receptor β-chain constant region for immunotherapy of T cell malignancies[J]. Nat Med.

[4] Gomes-Silva D, Srinivasan M, Sharma S (2017). CD7-edited T cells expressing a CD7-specific CAR for the therapy of T-cell malignancies[J]. Blood.

[5] Mamonkin M, Rouce RH, Tashiro H (2015). A T-cell-directed chimeric antigen receptor for the selective treatment of T-cell malignancies[J]. Blood.

[6] Cooper ML, DiPersio JF (2019). Chimeric antigen receptor T cells (CAR-T) for the treatment of T-cell malignancies[J]. Best Pract Res Clin Haematol.

[7] Wei W, Ma H, Yang D (2023). SECTM1-based CAR T cells enriched with CD7-low/negative subsets exhibit efficacy in CD7-positive malignancies[J]. Blood Adv.

[8] Png YT, Vinanica N, Kamiya T (2017). Blockade of CD7 expression in T cells for effective chimeric antigen receptor targeting of T-cell malignancies[J]. Blood Adv.

[9] Cooper ML, Choi J, Staser K (2018). An “off-the-shelf” fratricide-resistant CAR-T for the treatment of T cell hematologic malignancies[J]. Leukemia.

[10] Hu Y, Zhou Y, Zhang M (2022). Genetically modified CD7-targeting allogeneic CAR-T cell therapy with enhanced efficacy for relapsed/refractory CD7-positive hematological malignancies: a phase I clinical study[J]. Cell Res.

[11] Xie L, Gu R, Yang X (2023). Universal Anti-CD7 CAR-T cells targeting T-ALL and functional analysis of CD7 antigen on T/CAR-T cells[J]. Hum Gene Ther.

[12] Pan J, Tan Y, Wang G (2021). Donor-derived CD7 chimeric antigen receptor T cells for T-cell acute lymphoblastic leukemia: First-in-human, phase I trial[J]. J Clin Oncol.

[13] Lu P, Liu Y, Yang J (2022). Naturally selected CD7 CAR-T therapy without genetic manipulations for T-ALL/LBL: first-in-human phase 1 clinical trial[J]. Blood.

[14] Gonçalves CM, Henriques SN, Santos RF (2018). CD6, a Rheostat-type signalosome that tunes T cell activation[J]. Front Immunol.

[15] Kane LP, Lin J, Weiss A (2002). It's all Rel-ative: NF-kappaB and CD28 costimulation of T-cell activation[J]. Trends Immunol.

[16] Park SG, Schulze-Luehrman J, Hayden MS (2009). The kinase PDK1 integrates T cell antigen receptor and CD28 coreceptor signaling to induce NF-kappaB and activate T cells[J]. Nat Immunol.

[17] Yan X, Xu W, Yao H (2026). Activation of T cell-intrinsic p53 by acetylation elicits antitumor immunity to boost cancer immunotherapy[J]. Cancer Discov.

[18] Banerjee A, Thyagarajan K, Chatterjee S (2016). Lack of p53 augments antitumor functions in cytolytic T cells[J]. Cancer Res.

[19] Kastenhuber ER, Lowe SW (2017). Putting p53 in context[J]. Cell.

[20] Ghorashian S, Kramer AM, Onuoha S (2019). Enhanced CAR T cell expansion and prolonged persistence in pediatric patients with ALL treated with a low-affinity CD19 CAR[J]. Nat Med.

